# Supplementary material for: Asymmetric chromatin retention and nuclear envelopes separate chromosomes in fused cells in vivo
Source: Commun Biol. 2022 Sep 19;5:953. doi: 10.1038/s42003-022-03874-z (PMC9485224; doi:10.1038/s42003-022-03874-z)
Supplement: Supplementary file 2 — Supplementary Information [file 42003_2022_3874_MOESM2_ESM.pdf]

Supplementary Figures

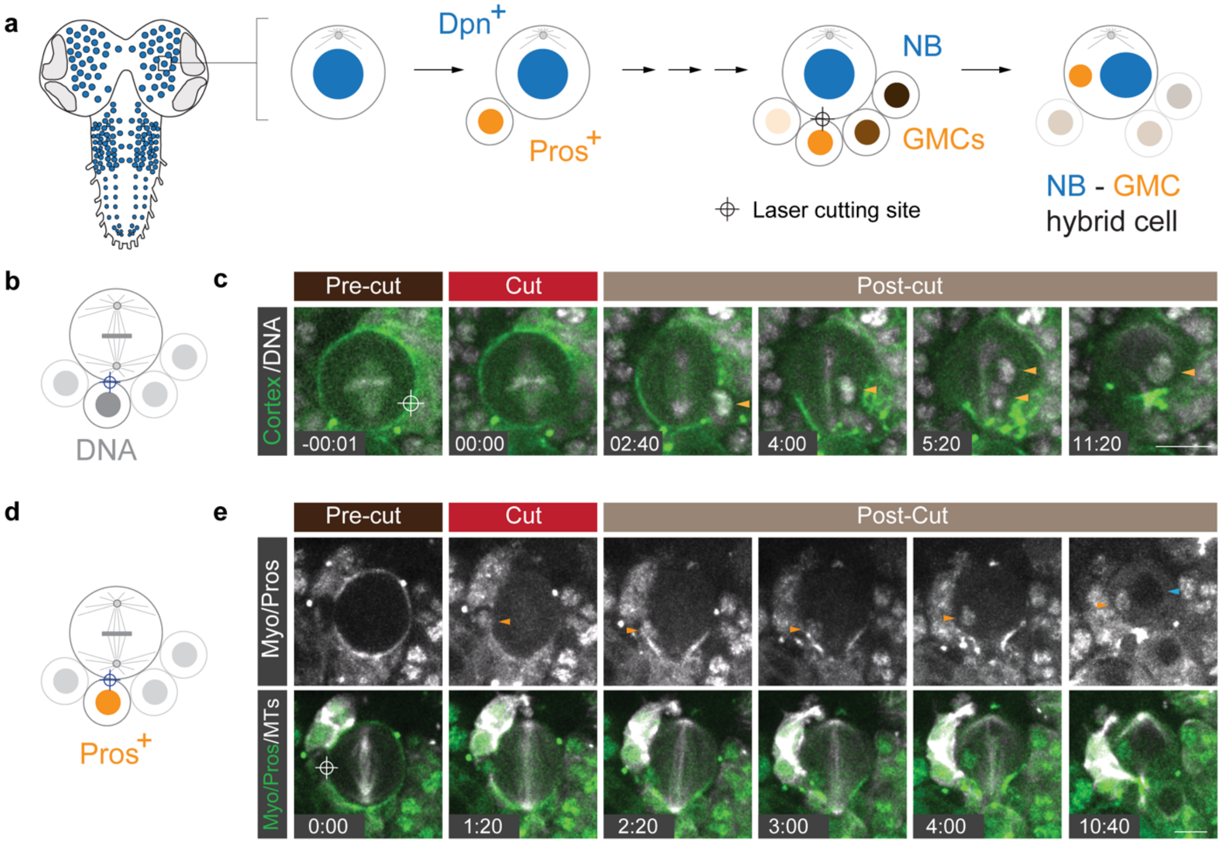

Supplementary Figure 1: Acute induced NB – GMC fusion in *Drosophila* larval brains.

(a) Schematic representation of a third instar *Drosophila* larval brain and neural stem cell division mode. Neural stem cells (neuroblasts (NBs); blue circles) divide asymmetrically, generating a Prospero-positive, differentiating ganglion mother cell (GMC; Pros$^+$) while self-renewing the Dpn$^+$ neuroblast. GMCs predominantly cluster on the NB's basal side and can be identified based on size and Prospero expression. Age differences of Pros$^+$ GMCs is indicated with different colors. Cutting sites (indicated with a crosshair) were chosen based on proximity to the NB and clearly identifiable cell boundaries. Acute NB – GMC fusion can result in hybrid cells containing two molecularly distinct nuclei. **(b)** Experimental outline and **(c)** representative example of a

metaphase wild type neuroblast, expressing the cell cortex marker Sqh::EGFP (green), the mitotic spindle marker cherry::Jupiter (white) and the chromatin marker His2A::RFP (white). The orange arrowhead labels a GMC nucleus moving into the NB. This hybrid cell successfully completes cytokinesis. **(d)** Schematic and **(e)** representative example of a wild type early anaphase neuroblast expressing Sqh::EGFP (white; top, green; bottom), cherry::Jupiter (white) and Pros::EGFP (white). The orange arrowhead highlights a $Pros^+$ GMC nucleus moving into the neuroblast. The blue arrowhead highlights the $Pros^-$ NB nucleus. Cytokinesis completes, creating a hybrid cell containing a $Pros^+$ and $Pros^-$ nucleus. Time in mins:secs; scale bar: 10μm.

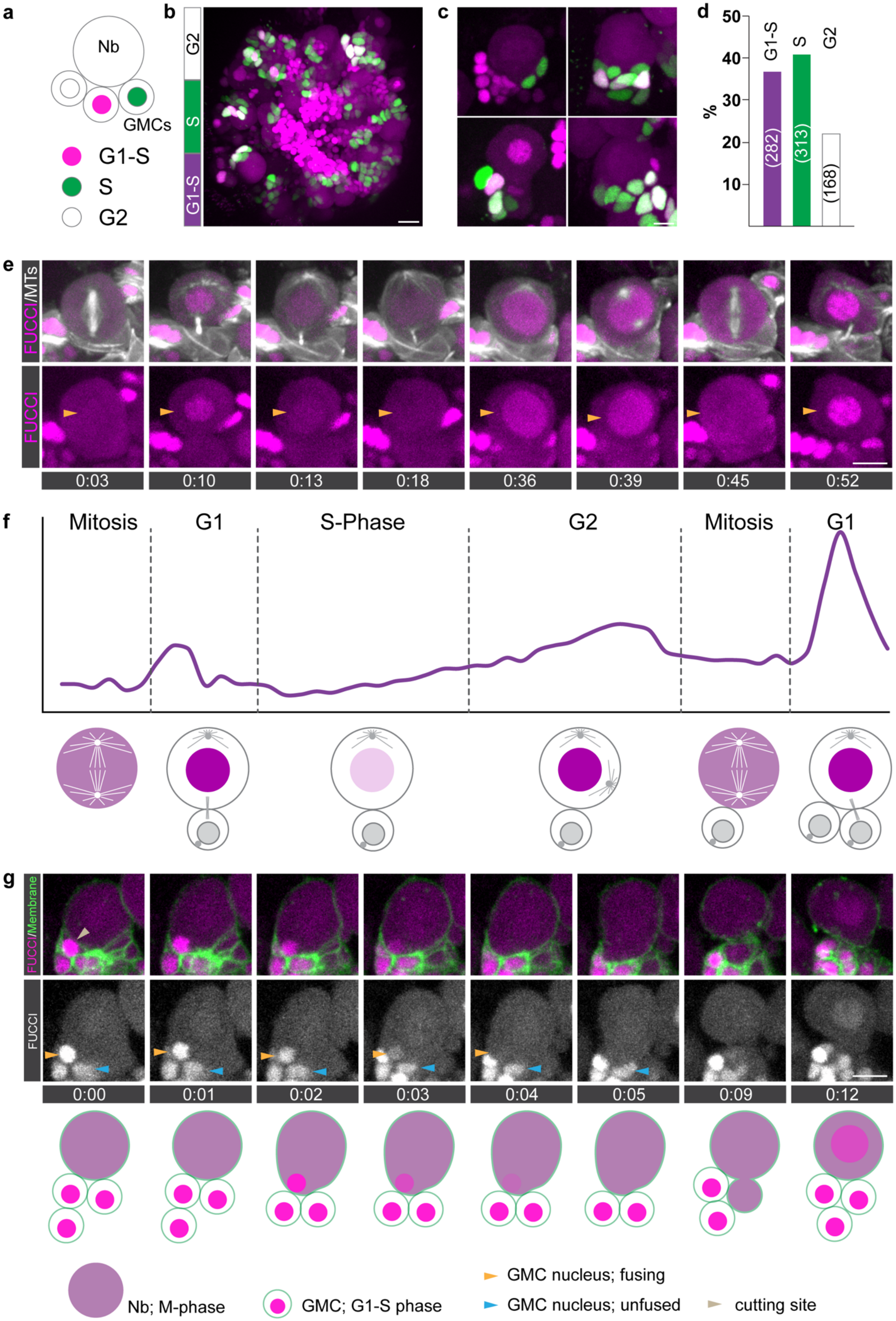
a
Nb
GMCs
G1-S
S
G2
b
G2
S
G1-S
c
d
50
40
30
20
10
%
G1-S
S
G2
(282)
(313)
(168)
e
FUCCI/MTs
FUCCI
0:03
0:10
0:13
0:18
0:36
0:39
0:45
0:52
f
Mitosis
G1
S-Phase
G2
Mitosis
G1
g
FUCCI/Membrane
FUCCI
0:00
0:01
0:02
0:03
0:04
0:05
0:09
0:12
Nb; M-phase
GMC; G1-S phase
GMC nucleus; fusing
GMC nucleus; unfused
cutting site

Supplementary Figure 2: Non-mitotic GMCs adjust its cell cycle to the parental NB after induced fusion.

(a) Fly-FUCCI was used to determine the cell cycle stage of GMCs/INPs in contact with neuroblasts. G1-S phase cells appear in magenta, S-phase cells in green and G2 cells in white (overlap between magenta and green). **(b)** Representative third instar brain and **(c)** example neuroblast lineages with neighboring GMCs/INPs. **(d)** Quantification of cell cycle stages. Only NB progeny in close vicinity to the parental NB were counted. **(e)** Wild type neuroblast expressing the Fly-FUCCI marker UAS-GFP.E2f1.1-230 together with the spindle marker cherry::Jupiter to distinguish between mitosis, G1-S, S- and G2-phase. Orange arrowheads indicate intensity changes of the cell cycle marker in the NB nucleus. **(f)** Intensity profile of UAS-GFP.E2f1.1-230 at different cell cycle stages. **(g)** Wild type neuroblast was fused with a neighboring G1-S phase GMC (orange arrowhead). Both cells express the Fly-FUCCI marker UAS-GFP.E2f1.1-230 and the membrane marker mCherry::CAAX. A neighboring GMC was labelled with a blue arrowhead. Schematic is shown below the image sequence. Time in hrs:mins; Scale bar is 5 μm.

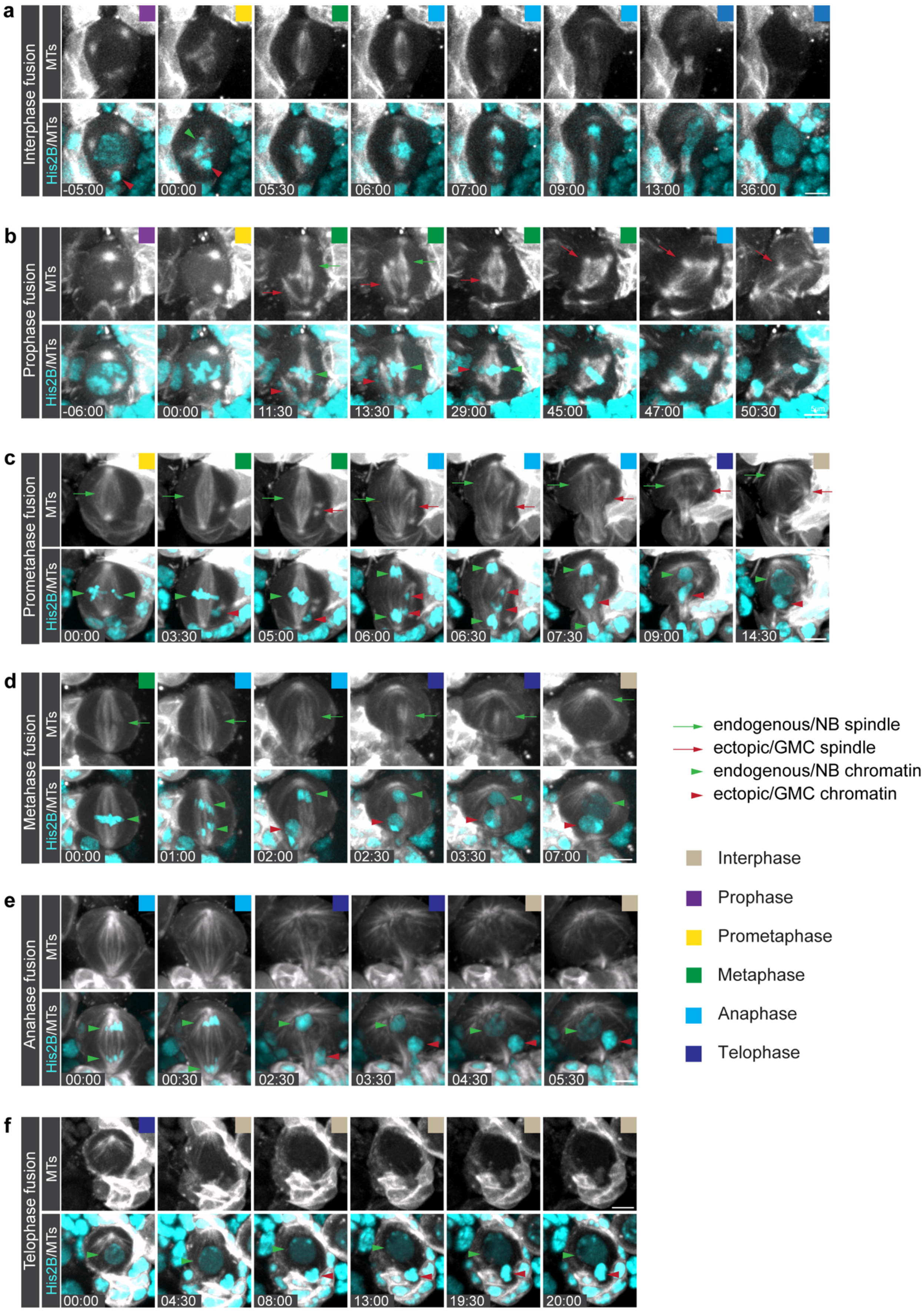
a
Interphase fusion
MTs
His2B/MTs
-05:00
00:00
05:30
06:00
07:00
09:00
13:00
36:00
b
Prophase fusion
MTs
His2B/MTs
-06:00
00:00
11:30
13:30
29:00
45:00
47:00
50:30
c
Prometahase fusion
MTs
His2B/MTs
00:00
03:30
05:00
06:00
06:30
07:30
09:00
14:30
d
Metahase fusion
MTs
His2B/MTs
00:00
01:00
02:00
02:30
03:30
07:00
e
Anahase fusion
MTs
His2B/MTs
00:00
00:30
02:30
03:30
04:30
05:30
f
Telophase fusion
MTs
His2B/MTs
00:00
04:30
08:00
13:00
19:30
20:00
endogenous/NB spindle
ectopic/GMC spindle
endogenous/NB chromatin
ectopic/GMC chromatin
Interphase
Prophase
Prometaphase
Metaphase
Anaphase
Telophase

Supplementary Figure 3: Double spindle formation in hybrid cells is cell cycle dependent. Representative images of wild type neuroblasts fused with GMCs in **(a)** interphase, **(b)** prophase, **(c)** prometaphase, **(d)** metaphase, **(e)** anaphase and **(f)** telophase. Chromatin is labelled with His2A::GFP (cyan) and microtubules with cherry::Jupiter (white). Green arrows and arrowheads label the NB spindle and chromatin. Red arrows and arrowheads label the GMC spindle and chromatin. Colored boxes indicated cell cycle stage. Time in mins:secs. Scale bar is 5 μm.

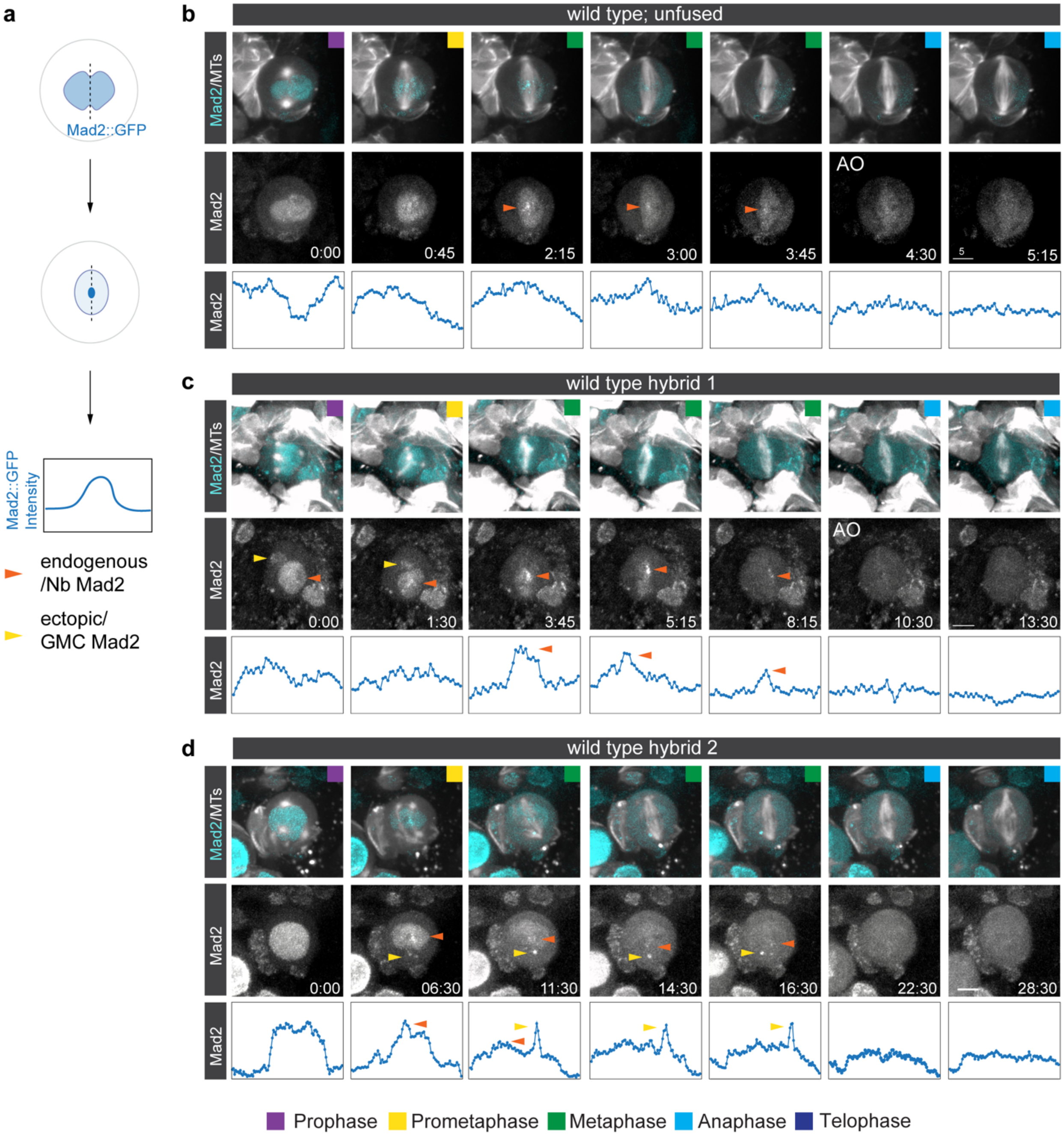

Supplementary Figure 4: Localized Mad2 disappears shortly before anaphase onset in hybrid cells.

(a) Schematic illustrating Mad2::GFP signal intensity measurements. Representative **(b)** unfused wild type neuroblast or **(c, d)** hybrid wild type cells expressing Mad2::GFP (top, cyan;

bottom, white) and cherry::Jupiter (top, white). Intensity plots are shown below the images. Red and yellow arrowheads highlight NB and GMC Mad2 signal, respectively. Colored boxes indicated cell cycle stage. Time in mins:secs. Scale bar is 5 μm.

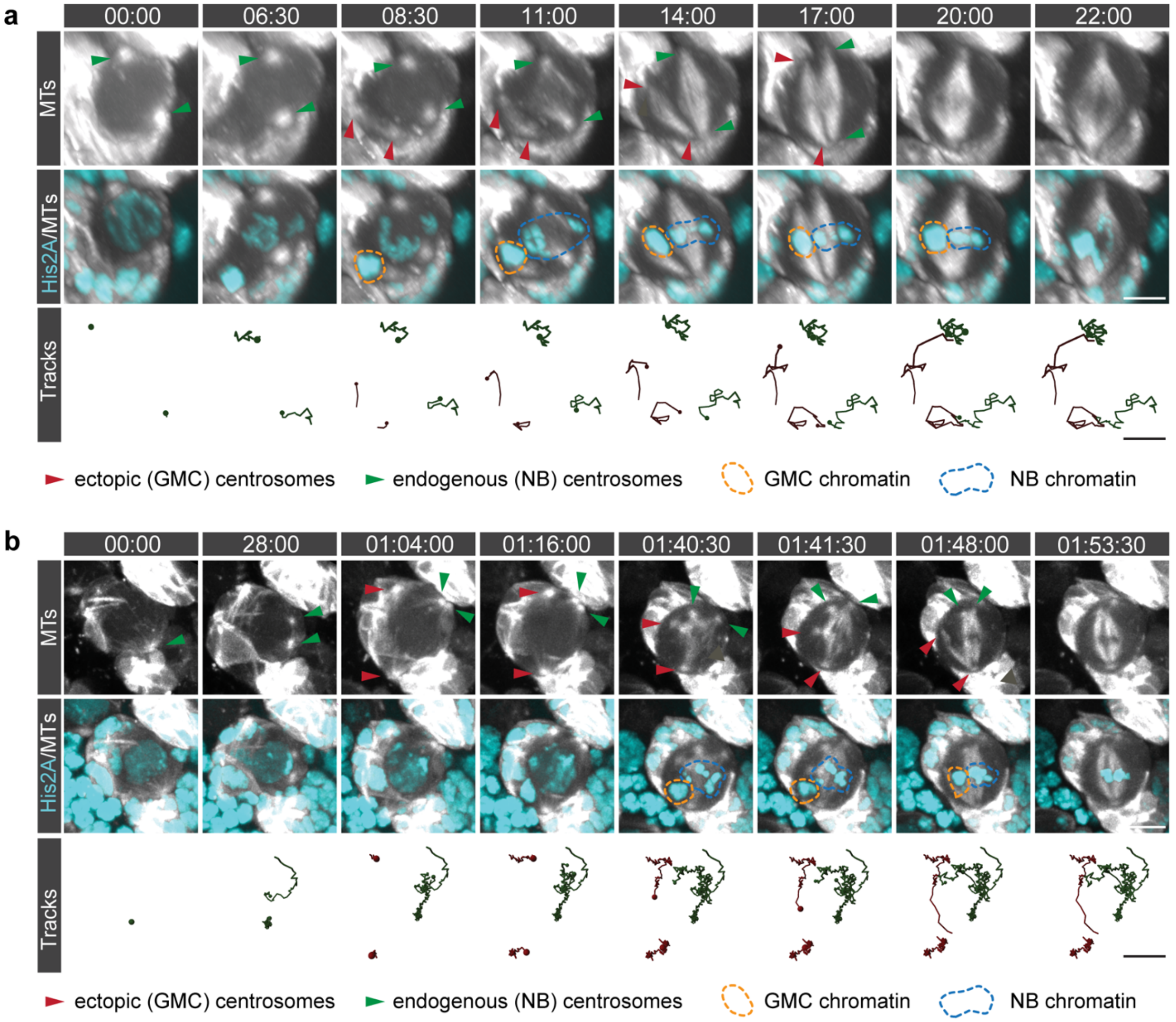

Supplementary Figure 5: Parallel or interconnected spindles separate endogenous from ectopic chromatin.

Additional example of a wild type hybrid cell forming a parallel **(a)** or interconnected **(b)** spindle. Hybrid cells all express the chromatin marker His2A::GFP (cyan) and the MT marker cherry::Jupiter. Centrosome tracks are shown underneath the snapshots. Green arrowheads label endogenous, NB-derived centrosomes. Brown and red arrowheads highlight ectopic, GMC-

derived centrosomes. NB- and GMC-derived chromatin are outlined with a blue and orange dashed line, respectively. Time in mins:secs; scale bar: 10μm.

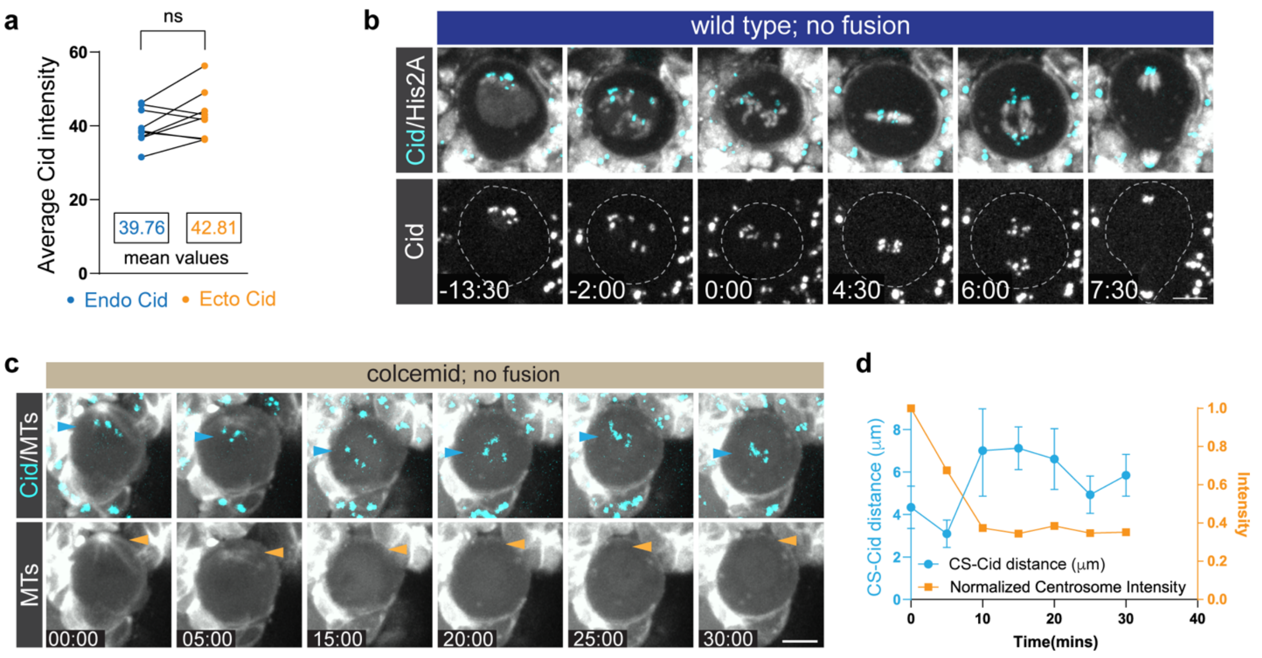

Supplementary Figure 6: Microtubule-dependent, asymmetric chromatin-centrosome attachments retain chromosomes close to the apical neuroblast cortex during interphase.

(a) CID intensity measurements of endogenous and ectopic CID in NB-GMC hybrid cells (p-value, 0.1294). **(b)** Representative wild type neuroblast expressing the canonical histone marker His2A::GFP (white), EGFP::Cid (cyan; top, white; below) and cherry::CAAX (white; top panel). The white dashed line highlights the cell outline. **(c)** Representative example of a wild type neuroblast expressing EGFP::Cid (cyan) and the microtubule marker cherry::Jupiter treated with colcemid. Blue and yellow arrowheads highlight the position of Cid clusters and the disappearing apical MTOC, respectively. **(d)** MTOC intensity and Cid location measurements for the cell shown in (c). Time in mins:secs. Scale bar is 5 μm.

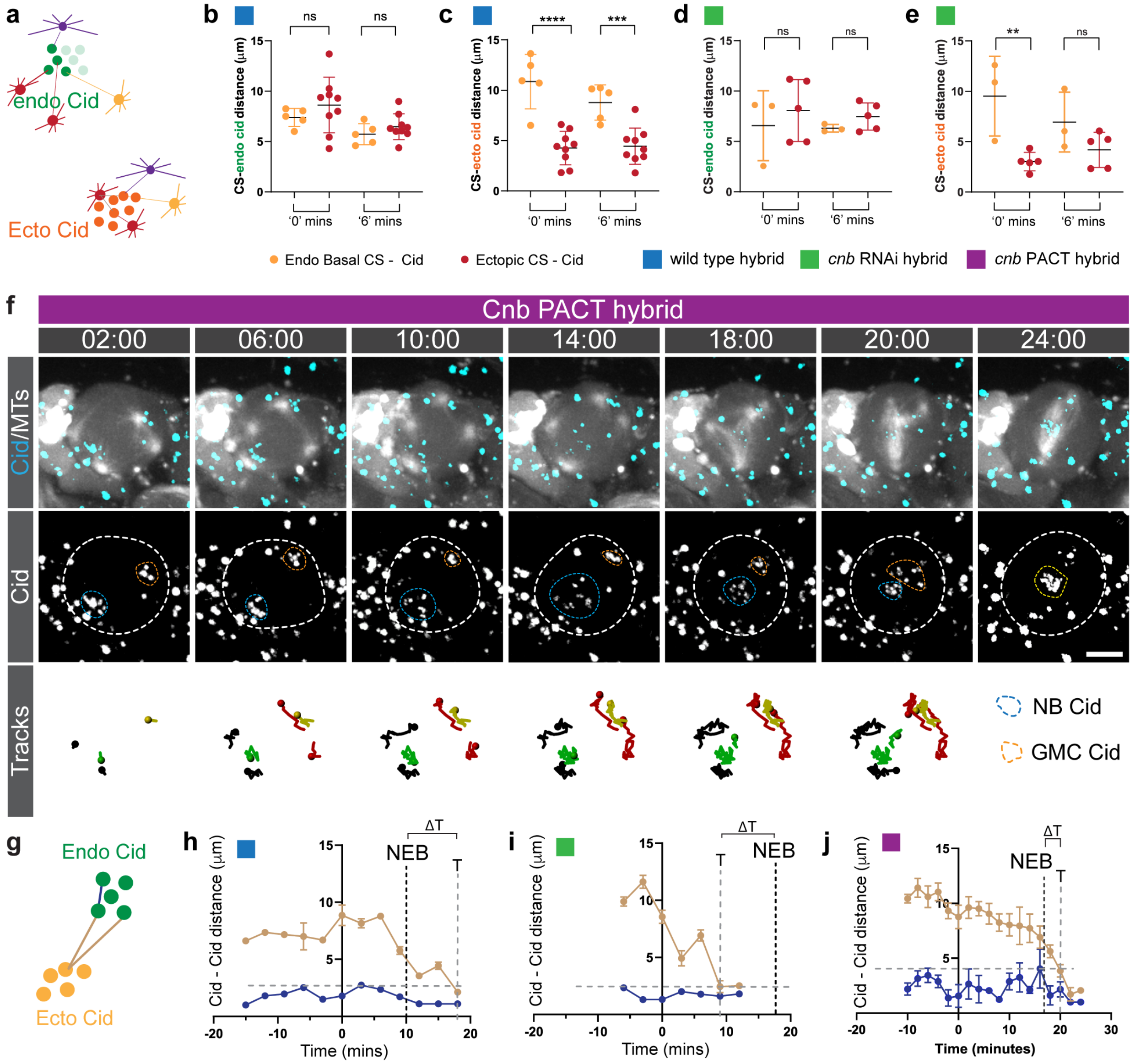

Supplementary Figure 7: Asymmetric MTOCs contribute to the separation of endogenous and ectopic chromatin in NB – GMC hybrid cells.

(a) The distance between endogenous (NB-derived; green) or ectopic (GMC-derived; orange) Cid was measured in relation to the NB's apical (magenta), basal (yellow) and the ectopic (red) GMC MTOCs. **(b)** Averaged distance of basal centrosomes to *endogenous* Cid in wild type or **(d)** *cnb* RNAi expressing neuroblasts. **(c)** Averaged distance of basal centrosomes to *ectopic* Cid in wild type or **(e)** *cnb* RNAi expressing neuroblasts. Measurements are plotted for '0' mins (ectopic centrosome maturation in wild type and *cnb* RNAi expressing hybrids) and 6 mins thereafter ('6').

(f) Representative image sequence of a cnb PACT expressing hybrid cell. Cell boundaries are outlined with the white dotted line. Endogenous and ectopic chromatin are highlighted with blue and orange dotted lines, respectively. Cid clusters that cannot be traced back to the Nb or GMC are highlighted with a yellow dotted line. MTOC and Cid cluster tracks are shown below the image sequence. **(g)** The distance between NB and GMC-derived Cid clusters was measured over time and representative examples are plotted (magenta lines) for **(h)** wild type, **(i)** cnb RNAi and **(j)** cnb PACT hybrid cells. The distance between NB-derived Cid clusters was used as a baseline (dark blue lines). Error bars correspond to SDs. Figure (b-e) two-sided unpaired t-test. ns; no significance. ** $p<0.01$, ***$p<0.001$, ****$p<0.0001$. Time in mins:secs. Scale bar is 5 μm.

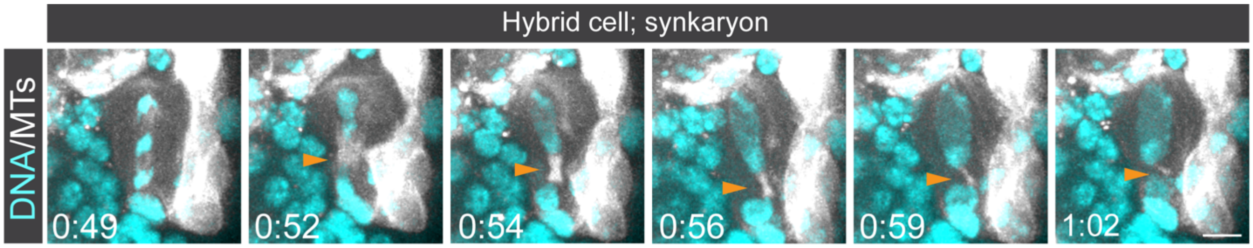

Supplementary Figure 8: Hybrid cells form synkaryons through fusion of the NB and GMC nucleus.

Same hybrid cell as shown in Figure 7c (bottom row), expressing the chromatin marker His2A::GFP (cyan) and the microtubule marker cherry::Jupiter (white). The hybrid cell forms a midbody – highlighted with the orange arrowhead – indicating completion of cytokinesis. Synkaryon formation is a consequence of nuclear fusion. Time in hrs:mins. Scale bar is 5 μm.
