# Supplementary material for: Asymmetric chromatin retention and nuclear envelopes separate chromosomes in fused cells in vivo
Source: Commun Biol. 2022 Sep 19;5:953. doi: 10.1038/s42003-022-03874-z (PMC9485224; doi:10.1038/s42003-022-03874-z)
Supplement: Supplementary file 3 — Description of Additional Supplementary Files [file 42003_2022_3874_MOESM3_ESM.pdf]

Description of Additional Supplementary Files

File name: Supplementary Data 1

Descriptions: Statistics summary. Complete statistical information for the shown data.

File name: Supplementary Data 2

Descriptions: Measured raw values for the shown figures.

File name: Supplementary Movie 1

Descriptions: Wild type hybrid cell derived from a neuroblast – GMC fusion *in vivo*, expressing the cell cycle marker *UAS-mRFP1.NLS.CycB.1* in cyan and a membrane marker in green. The NB was in early mitosis when fusion was induced and the GMC in G1-S phase. The orange arrow marks the GMC nucleus that entered the NB cytoplasm. Time scale is h:mm:ss and the scale bar is 4 µm.

File name: Supplementary Movie 2

Descriptions: Wild type control (unfused) neuroblast expressing the microtubule binding protein Cherry::Jupiter (white) and the canonical Histone marker His2A::GFP (cyan). Time scale is h:mm:ss and the scale bar is 5 µm.

File name: Supplementary Movie 3

Descriptions: Wild type hybrid cell derived from a neuroblast – GMC fusion *in vivo*, expressing the canonical Histone marker His2A::GFP (white in single channel; cyan in merge) and the microtubule binding protein Cherry::Jupiter (white). The blue and orange arrows mark

endogenous and ectopic chromatin, respectively. Time scale is h:mm:ss and the scale bar is 3 μm.

File name: Supplementary Movie 4

Descriptions: Wild type hybrid cell derived from a neuroblast – GMC fusion *in vivo*, expressing the canonical Histone marker His2A::GFP (white in single channel; cyan in merge) and the microtubule binding protein Cherry::Jupiter (white). The blue and orange arrows mark endogenous and ectopic chromatin, respectively. Time scale is h:mm:ss and the scale bar is 2 μm.

File name: Supplementary Movie 5

Descriptions: Wild type hybrid cell, expressing the microtubule binding protein Cherry::Jupiter (white). The hybrid cell forms a 'II'-type spindle. The blue and red arrows mark the NB and GMC spindle, respectively. Time scale is h:mm:ss:ms and the scale bar is 5 μm.

File name: Supplementary Movie 6

Descriptions: Unfused *asl* mutant neuroblast, expressing the canonical Histone marker His2A::GFP (cyan) and the microtubule binding protein Cherry::Jupiter (white). Time scale is h:mm:ss:ms and the scale bar is 5 μm.

File name: Supplementary Movie 7

Descriptions: *asl* mutant hybrid cell expressing the canonical Histone marker His2A::GFP (cyan) and the microtubule binding protein Cherry::Jupiter (white). Green and red arrows highlight the NB and GMC chromatin, respectively. This hybrid cell was derived from fusing two GMCs with one NB. Time scale is h:mm:ss:ms and the scale bar is 5 μm.

File name: Supplementary Movie 8

Descriptions: Wild type control (unfused) neuroblast, expressing the microtubule binding protein Cherry::Jupiter (white) and the centromere-specific H3 variant EGFP::Cid (Cyan). Purple and yellow arrows point to the apical and basal centrosome, respectively. The blue arrow refers to moving Cid clusters. Time scale is h:mm:ss:ms and the scale bar is 1 µm.

File name: Supplementary Movie 9

Descriptions: Wild type control (unfused) neuroblast, expressing the membrane marker mCherry::CAAX (white), the canonical Histone marker His2A::GFP (white) and EGFP::Cid (Cyan). The green arrow points to Cid clusters. Time scale is h:mm:ss:ms and the scale bar is 5 µm.

File name: Supplementary Movie 10

Descriptions: Wild type control (unfused) neuroblast exposed to the microtubule depolymerizing drug Colcemid, expressing the microtubule binding protein Cherry::Jupiter (white) and EGFP::Cid (Cyan). The yellow arrow points to the apical centrosome, the blue arrow to Cid clusters. Time scale is h:mm:ss:ms and the scale bar is 1 µm.

File name: Supplementary Movie 11

Descriptions: *cnb* RNAi expressing (unfused) neuroblast, co-expressing the microtubule binding protein Cherry::Jupiter (white) and EGFP::Cid (Cyan). The green and red arrows point to the apical MTOC and Cid cluster, respectively. h:mm:ss:ms and the scale bar is 3 µm.

File name: Supplementary Movie 12

Descriptions: Wild type hybrid cells expressing the microtubule binding protein Cherry::Jupiter (white) and EGFP::Cid (white in single channel; Cyan in merge). The blue and orange arrow

highlights endogenous and ectopic Cid clusters, respectively. Time scale is h:mm:ss:ms and the scale bar is 1 µm.

File name: Supplementary Movie 13

Descriptions: *cnb* RNAi expressing hybrid cell, expressing the microtubule binding protein Cherry::Jupiter (white) and EGFP::Cid (cyan). NB and GMC Cid are highlighted with a blue and orange circle respectively. The yellow circle labels merged Cid clusters. The second movie shows segmented and color-coded (NB Cid; blue, GMC Cid; orange, merged Cid; green) Cid clusters. Cid signal outside of the hybrid cell was removed for better visibility. Time scale is h:mm:ss:ms and the scale bar is 1 µm.

File name: Supplementary Movie 14

Descriptions: Wild type hybrid cell expressing the nuclear envelope (NE) marker Lamin::GFP (cyan in merge; white in single channel) and the microtubule binding protein Cherry::Jupiter (white in merge). Green and red arrows highlight NB and GMC NE, respectively. Time scale is h:mm:ss:ms and the scale bar is 5 µm.

File name: Supplementary Movie 15

Descriptions: Wild type hybrid cell, expressing the canonical Histone marker His2A::GFP (white). The blue and orange arrow highlights endogenous and ectopic chromosomes, respectively. The magenta arrowhead highlights the fate of missegregated chromosomes. This hybrid cell forms a heterokaryon. Time scale is h:mm:ss:ms and the scale bar is 1 µm.
