# Supplementary material for: Asymmetric chromatin retention and nuclear envelopes separate chromosomes in fused cells in vivo
Source: Commun Biol. 2022 Sep 19;5:953. doi: 10.1038/s42003-022-03874-z (PMC9485224; doi:10.1038/s42003-022-03874-z)
Supplement: Supplementary file 21 — Reporting Summary [file 42003_2022_3874_MOESM21_ESM.pdf]

nature portfolio

Corresponding author(s): Clemens Cabernard

Last updated by author(s): 2022/05/27

Reporting Summary

Nature Portfolio wishes to improve the reproducibility of the work that we publish. This form provides structure for consistency and transparency in reporting. For further information on Nature Portfolio policies, see our Editorial Policies and the Editorial Policy Checklist.

Statistics

For all statistical analyses, confirm that the following items are present in the figure legend, table legend, main text, or Methods section.

n/a	Confirmed	
☐	☒	The exact sample size (n) for each experimental group/condition, given as a discrete number and unit of measurement
☐	☒	A statement on whether measurements were taken from distinct samples or whether the same sample was measured repeatedly
☐	☒	The statistical test(s) used AND whether they are one- or two-sided<br>*Only common tests should be described solely by name; describe more complex techniques in the Methods section.*
☒	☐	A description of all covariates tested
☒	☐	A description of any assumptions or corrections, such as tests of normality and adjustment for multiple comparisons
☐	☒	A full description of the statistical parameters including central tendency (e.g. means) or other basic estimates (e.g. regression coefficient) AND variation (e.g. standard deviation) or associated estimates of uncertainty (e.g. confidence intervals)
☐	☒	For null hypothesis testing, the test statistic (e.g. F, t, r) with confidence intervals, effect sizes, degrees of freedom and P value noted<br>*Give P values as exact values whenever suitable.*
☒	☐	For Bayesian analysis, information on the choice of priors and Markov chain Monte Carlo settings
☒	☐	For hierarchical and complex designs, identification of the appropriate level for tests and full reporting of outcomes
☒	☐	Estimates of effect sizes (e.g. Cohen's d, Pearson's r), indicating how they were calculated

Our web collection on statistics for biologists contains articles on many of the points above.

Software and code

Policy information about availability of computer code

Data collection	Imaging data was collected with Slidebook acquisition software.
Data analysis	Imaging data was analyzed in Imaris x64 8.3.1 and image J.

For manuscripts utilizing custom algorithms or software that are central to the research but not yet described in published literature, software must be made available to editors and reviewers. We strongly encourage code deposition in a community repository (e.g. GitHub). See the Nature Portfolio guidelines for submitting code & software for further information.

Data

Policy information about availability of data

All manuscripts must include a data availability statement. This statement should provide the following information, where applicable:

- Accession codes, unique identifiers, or web links for publicly available datasets
- A description of any restrictions on data availability
- For clinical datasets or third party data, please ensure that the statement adheres to our policy

The authors declare that all data supporting the findings of this study are available within the paper and its supplementary files. Source data are available upon request

Field-specific reporting

Please select the one below that is the best fit for your research. If you are not sure, read the appropriate sections before making your selection.

☒ Life sciences ☐ Behavioural & social sciences ☐ Ecological, evolutionary & environmental sciences

For a reference copy of the document with all sections, see nature.com/documents/nr-reporting-summary-flat.pdf

Life sciences study design

All studies must disclose on these points even when the disclosure is negative.

Sample size	Sample size calculations were not performed. Sample sizes were chosen empirically and based on what was technically possible.
Data exclusions	Only data of insufficient quality (signal/noise too high, pixel count too low, lack of temporal resolution or obstructing signals).
Replication	All measurements were obtained from distinct samples and several independent experiments. The main findings could be confirmed accross all experiments.
Randomization	Organisms (Drosophila) were not randomized but for each condition, growth temperature and time of development before experiment were controlled.
Blinding	Blinding does not apply here because Drosophila crosses and following experiment can not be randomized.

Reporting for specific materials, systems and methods

We require information from authors about some types of materials, experimental systems and methods used in many studies. Here, indicate whether each material, system or method listed is relevant to your study. If you are not sure if a list item applies to your research, read the appropriate section before selecting a response.

Materials & experimental systems

n/a	Involved in the study	
☐	☒	Antibodies
☒	☐	Eukaryotic cell lines
☒	☐	Palaeontology and archaeology
☐	☒	Animals and other organisms
☒	☐	Human research participants
☒	☐	Clinical data
☒	☐	Dual use research of concern

Methods

n/a	Involved in the study	
☒	☐	ChIP-seq
☒	☐	Flow cytometry
☒	☐	MRI-based neuroimaging

Antibodies

Antibodies used	The following primary antibodies were used for this study: mouse anti-a-Tub (DM1A, Sigma; 1:2500; Cat# T6199), rabbit anti-Cid (1:500; Activemotif; Cat#39719). Secondary antibodies were from Invitrogen.
Validation	with endogenously tagged proteins (Cid::EGFP), Tub::cherry.

Animals and other organisms

Policy information about studies involving animals; ARRIVE guidelines recommended for reporting animal research

Laboratory animals	Drosophila melanogaster was used for this work. Adults were crossed and progeny at the larval stage were dissected to collect the central nervous system.<br>The list and the origin of the various Drosophila strains (Bloomington, VDRC, other labs, generated for this work) are describe in Method section.
Wild animals	No wild animals were used in this study.
Field-collected samples	No field collected samples are used in this study.
Ethics oversight	No ethical approval was needed to conduct this study

Note that full information on the approval of the study protocol must also be provided in the manuscript.
